# Synthesis of lipophilic tyrosyl esters derivatives and assessment of their antimicrobial and antileishmania activities

**DOI:** 10.1186/1476-511X-11-13

**Published:** 2012-01-20

**Authors:** Imen Aissa, Rabiaa Manel Sghair, Mohamed Bouaziz, Dhafer Laouini, Sami Sayadi, Youssef Gargouri

**Affiliations:** 1 Laboratoire de Biochimie et de Génie Enzymatique des Lipases, Ecole Nationale d'Ingénieurs de Sfax (ENIS). Route de Soukra, BP 1173, 3038 Sfax, Université de Sfax,Tunisie; 2 Groupe Immunobiologie des Leishmanioses, Labratoire de Transmission, Contrôle et Immunobiologie des Infections (LTCII), Institut Pasteur de Tunis, 13, place Pasteur, B.P 74, 1002 Tunis-Belvédère, Tunisie; 3 Laboratoire des Bioprocédés, Centre de Biotechnologie de Sfax (CBS). BP 1177, 3018 Sfax, Université de Sfax, Tunisie

**Keywords:** Tyrosol, antioxidant, antimicrobial activity, leishmanicidal activity

## Abstract

**Background:**

Preparation of tyrosyl lipophilic derivatives was carried out as a response to the food, cosmetic and pharmaceutical industries' increasing demand for new lipophilic antioxidants.

**Results:**

A large series of tyrosyl esters (**TyC_2 _**to **TyC_18:1_**) with increasing lipophilicity was synthesized in a good yield using lipase from *Candida antarctica *(Novozyme 435). Spectroscopic analyses of purified esters showed that the tyrosol was esterified on the primary hydroxyl group. Synthetized compounds were evaluated for either their antimicrobial activity, by both diffusion well and minimal inhibition concentration (MIC) methods, or their antileishmanial activity against *Leishmania major *and *Leishmania infantum *parasite species.

Among all the tested compounds, our results showed that only **TyC_8_**, **TyC_10 _**and **TyC_12 _**exhibited antibacterial and antileishmanial activities. When MIC and IC_50 _values were plotted against the acyl chain length of each tyrosyl derivative, **TyC_10 _**showed a parabolic shape with a minimum value. This nonlinear dependency with the increase of the chain length indicates that biological activities are probably associated to the surfactant effectiveness of lipophilic derivatives.

**Conclusion:**

These results open up potential applications to use medium tyrosyl derivatives surfactants, antioxidants, antimicrobial and antileishmanial compounds in cosmetic, food and pharmaceutical industries.

## Background

Polyphenolic compounds produced by plants are of considerable interest, both as functional food ingredients and as nutraceuticals [1]. In addition to their antioxidant properties, several studies showed that phenolic compounds also have antimicrobial properties by denaturing proteins and inactivating enzymes [2,3]. Tyrosol [2-(4-hydroxyphenyl) ethanol] is a well-known monophenolic antioxidant present in large amount in olive oil and can be extracted from olive mill waste water [4]. Its efficiency was demonstrated in inhibiting the oxidation of cholesterol in LDL and preventing the modification of the apoproteic moiety [5]. Tyrosol has been also effective in inhibiting leukocyte 5-lipooxygenase [6] and protecting the Caco-2 intestinal mucosa cells against the cytostatic and cytotoxic effects produced by oxidized LDL [7]. Many other activities of tyrosol were described such as its ability to inhibit ADP-induced platelet aggregation [8], to significantly reduce the arrhythmic activity that occurs during myocardial ischemia and reperfusion [9], and to possess significant neuroprotective activities against glutamate-induced neurotoxicity in primary cultures of rat cortical cells and injury induced by 5-S-cysteinyl-dopamine *in vitro *[10]. Hence, lipophilic derivatives of tyrosol and, in particular, esters bearing acyl chains, exhibit a better affinity with lipophilic membrane constituents. For this reason and others, these compounds could be important for further application in pharmaceutical and cosmetic fields [11]. Some tyrosyl derivatives have been found in diverse natural sources e.g., the presence of its acetate was reported in virgin olive oil [12], and its lipophilic palmitate, stearate, and oleate esters were isolated from *Ligustrum ovalifolium *flowers [13], from the stem bark of *Buddleja cordata *[14], and from olive fruits [15], respectively. In addition, the use of some natural tyrosyl esters for antiaging and/or pharmacological applications has been of attracting interest in the past few years [16]. For all these reasons, growing attention has been devoted to the synthesis of tyrosyl esters derived from fatty acids. Short, medium and long chain derivatives of the tyrosol were synthesized by trans-esterification reactions using lipases [17,18]. Amphiphilic tyrosol derivatives display particularly interesting characteristics, resulting from the modification of molecular flexibility. The evaluation of antioxidant activity using Rancimat, FRAP and ABTS methods showed that tyrosyl esters are less active than free tyrosol [18]. Few data regarding the biological activities of tyrosyl esters have been reported. Fragoupoulou et al, [11] have shown that the monoacetylated tyrosol is two orders of magnitude more potent as anti-thrombic agent than tyrosol, and Ahn et al, [19] have reported that *p-*tyrosyl acetate reduces the cell viability of some cancer cell types better than tyrosol. Singh et al, [20] have synthesized several piperoyl-amino acides ester conjugates (chemical derivatives of alkaloid piperine) and have evaluated their antileishmanial activity *in vitro *and *in vivo*. They found that piperoyl-valine methyl ester showed the best activity against the amastigotes and a reduction of 24% in spleen parasitic burden *in vivo *assay with golden hamsters. However, there is no data, in the literature, showing the antileishmanial activity of tyrosyl esters derivatives.

In this purpose, we have synthetized a large series of tyrosyl fatty acid esters by direct esterification of tyrosol with different fatty acids using Novozyme 435 as catalyst and evaluated their anti-microbial activity against several pathogenic strains and their anti-leishmanial effects on both *Leishmania *(*L*) *major *and *L. infantum *strains.

## Results

### Preparation and Characterization of Tyrosyl Esters

**Figure 1:**
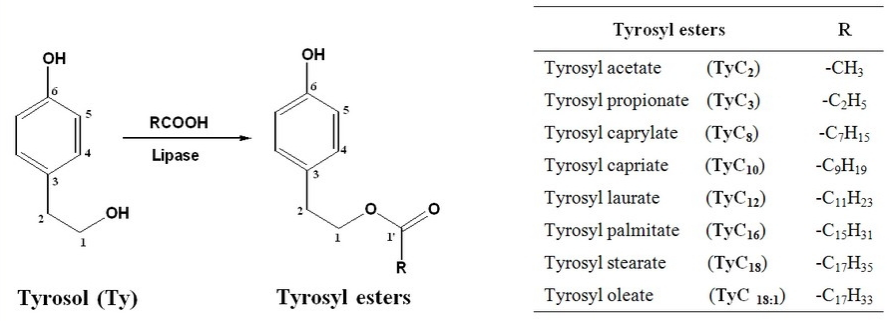
**Structure of Synthesized Compounds**.

A chemoselective procedure was used to synthetize lipophilic tyrosyl esters (**TyC_2 _**to **TyC_18:1_**) (Figure 1). Lipase from *C. antarctica *has been used as a catalyst for the esterification reactions. The conversion yields calculated after 72 h are respectively: **TyC_2_**: 99.74%, **TyC_3_**: 95.93%, **TyC_8_**: 85.55%, **TyC_10_**: 75.42%, **TyC_12_**: 73.33%, **TyC_16_**: 69.95%, **TyC_18_**: 66.95% and **TyC_18:1_**: 57%. As shown, highest ester synthesis yields were obtained when using short acyl chains ester (**TyC_2 _**and **TyC_3_**). For medium and long chain esters (**TyC_8 _**to **TyC_18:1_**), the conversion yield decrease with the increasing of the acyl chain length. The same results were obtained by Mateos et al, [18] for the synthesis of tyrosyl esters by a transesterifcation reaction, but the rate of the reaction is higher than the esterification procedure. This can be attributed to water production during the esterification reaction, which can promote the hydrolysis of formed ester. However, during the transesterification reaction, the reaction temperature favorites the elimination of methanol, produced as co product, by evaporation. This kind of process can contribute to shift the reaction equilibrium to the synthesis of ester [21].

The fatty acid unsaturation seems to affect the synthesis yield. In fact, the conversion yield obtained with the tyrosyl stearate, 66.95%; decreases to 57% when using the oleic acid to synthesize the tyrosyl oleate. Our observations are in agreement with those described by Selmi et al. [22] when synthesizing several triacylglycerol esters using immobilized *Rhizomucor miehei *lipase. These authors concluded that the increase of the unsaturation number is responsible for the lower rate of triacylglycerols synthesis [22].

Tyrosyl esters were characterized by NMR. As shown in Table 1 there are differences of 0.61 and 0.21 ppm, respectively, for the chemical shifts (δ) of H_1 _and H_2 _with respect to the same values in free tyrosol. For the aromatic protons, these differences are very weak but still appreciable (0.03 and 0.08 ppm). Similar deshielding effects can be observed from ^13^C data (Table 2), although the expected β-shielding effect is evident for C_2 _in each ester.

**Table 1:** ^1^H NMR Data (300 MHz, CDCL_3_) for Compounds **Ty **to **TyC_18:1_**

	(Ty)	(TyC_2_)	(TyC_3_)	(TyC_8_)	(TyC_10_)	(TyC_12_)	(TyC_16_)	(TyC_18_)	(TyC_18: 1_)
1	3.72 (t)	4.26 (t)	4.26 (t)	4.28 (t)	4.24 (t)	4.25 (t)	4.31 (t)	4.23 (t)	4.23 (t)

2	2.71 (t)	2.87 (t)	2.87 (t)	2.87 (t)	2.85 (t)	2.85 (t)	2.92 (t)	2.85 (t)	2.82 (t)

4	7.11 (d) J_4,5 _= 8.4	7.10 (d) J_4,5_= 8.4	7.07 (d) J_4,5_= 8.4	7.08 (d) J_4,5_= 8.4	7.06 (d) J_4,5_= 8.4	7.04 (d) J_4,5_= 8.4	7.12 (d) J_4,5_= 8.4	7.07 (d) J_4,5_= 8.4	7.09 (d) J_4,5_= 8.4

5	6.79 (d) J_4,5_= 9	6.80 (d) J_4,5_= 9	6.80 (d) J_4,5_= 9	6.80 (d) J_4,5_= 9	6.79 J_4,5_= 9	6.80 (d) J_4,5_= 9	6.80 (d) J_4,5_= 9	6.80 (d) J_4,5_= 9	6.80 (d) J_4,5_= 9

6		5.50 (s)	5.50 (s)	5.50 (s)	5.50 (s)	5.50 (s)	6.00(s)	5.10 (s)	5.30 (s)

2'		2.06 (s)	2.32(q)	2.33(t)	2.29 (t)	2.30(t)	2.36(t)	2.30(t)	2.30(t)

3'			1.13 (t)	1.60(m)	1.59 (m)	1.60 (m)	1.66(m)	1.60 (m)	1.60 (m)

4'				1.28(m)	1.25 (m)	1.26 (m)	1.32 (m)	1.25 (m)	1.25 (m)

(CH_2_)_3_				1.28(m)	1.25 (m)	1.26 (m)	1.32 (m)	1.25 (m)	1.25 (m)

8'				0.9 (t)	1.25 (m)	1.26 (m)	1.32 (m)	1.25 (m)	2.00 (m)

9'					1.25 (m)	1.26 (m)	1.32 (m)	1.25 (m)	5.33 (m)

10'					0.88 (t)	1.26 (m)	1.32 (m)	1.25 (m)	5.33 (m)

11'						1.26 (m)	1.32 (m)	1.25 (m)	2.00 (m)

12'						0.88 (t)	1.32 (m)	1.25 (m)	1.25 (m)

13'							1.32 (m)	1.25 (m)	1.25 (m)

14'							1.32 (m)	1.25 (m)	1.25 (m)

(CH_2_)_n_							1.32 (m)	1.25 (m)	1.25 (m)

Me							0.94 (t)	0.87 (t)	0.87 (t)

**Table 2:** ^13^C NMR Chemical Shifts (ppm) (125

	(Ty)	(TyC_2_)	(TyC_3_)	(TyC_8_)	(TyC_10_)	(TyC_12_)	(TyC_16_)	(TyC_18_)	(TyC_18: 1_)
1	63.1	65.7	65.6	65.9	65.3	63.7	65.6	65.6	65.7

2	37.2	34.6	34.6	34.6	34.4	32.7	34.8	34.8	35.0

3	130.6	130.2	130.12	129.6	129.9	127.5	129.9	129.9	130.9

4	131.3	130.4	130.41	130.3	129.4	128.2	130.3	130.3	130.07

5	115.7	115.8	115.7	115.9	115.4	113.7	115.7	115.7	116.1

6	154.1	154.8	154.8	155.2	154.7	153.1	154.9	154.9	155.4

1'		171.9	174.1	175.4	174.7	173.1	174.9	174.4	174.6

2'		21.4	30.09	34.8	34.3	32.7	34.6	34.6	32.6

3'			28.04	25.3	31.9	23.2	25.3	25.34	25.7

4'				29.4-29.2	29.4-29.1	27.9-27.4	29.5-30.1	29.5-30.1	29.8-28.0

(CH_2_)_3_				29.4-29.2	29.4-29.1	27.9-27.4	29.5-30.1	29.5-30.1	29.8-28.0

8'				14.4	24.8	27.9-27.4	29.5-30.1	29.5-30.1	27.2

9'					22.6	27.9-27.4	29.5-30.1	29.5-30.1	129.8

10'					14.1	32.5	29.5-30.1	29.5-30.1	128.0

11'						21.0	29.5-30.1	29.5-30.1	27.2

12'						12.43	29.5-30.1	29.5-30.1	29.8-28.0

13'							29.5-30.1	29.5-30.1	29.8-28.0

14'							32.3	29.5-30.1	29.8-28.0

15'							23.1	29.5-30.1	29.8-28.0

16'							14.5	32.3	31.9

17'								23.09	23.4

18'								14.5	14.8

### Antimicrobial activity of tyrosyl derivatives

Tyrosol and its esters were investigated for their antimicrobial activity against several pathogenenic bacteria spp. Tyrosol showed no inhibition against all the bacteria tested in this study (Table 3). Among all esters tested, only medium chain tyrosyl derivatives (**TyC_8_**, **TyC_10 _and TyC_12_**) exhibited an antibacterial activity. Hence, at 20 mg/ml, **TyC_8 _**and **TyC_10 _**showed the highest inhibitory activity against *S. aureus, S. xylosus, B. cereus *and *B. flavum *whereas tyrosyl laurate (**TyC_12_**) exhibited the lowest antimicrobial activity against Staphylococcus strains.

**Table 3:** Inhibitory spectrum of tyrosol (**Ty**) and tyrosyl esters on Gram-positive and Gram-negative bacteria

Strain	Gram	Sensibility								
		**Ty**	**TyC**_**2**_	**TyC**_**3**_	**TyC**_**8**_	**TyC**_**10**_	**TyC**_**12**_	**TyC**_**16**_	**TyC**_**18**_	**TyC**_**18:1**_

*Bacillus cereus*	+	-	-	-	**+**	**++**	-	-	-	-

*Bacillus subtilis*	+	-	-	-	**-**	-	-	-	-	-

*Micrococcus luteus*	+	-	-	-	-	-	-	-	-	-

*Brevibacterium flavum*	+	-	-	-	**+**	**+**	-	-	-	-

*Enterococcus faecalis*	+	-	-	-	-	-	-	-	-	-

*Staphylococcus aureus*	+	-	-	-	**++**	**++**	**+**	-	-	-

*Staphylococcus xylosus*	+	-	-	-	**++**	**++**	**+**	-	-	-

*Staphylococcus epidermidis*	+	-	-	-	-	-	-	-	-	-

*Pseudomonas aerigenosa*	**-**	-	-	-	**+**	-	-	-	-	-

*Enterobacter cloacae*	**-**	-	-	-	**-**	-	-	-	-	-

*Klebsielle pneumoniae*	**-**	-	-	-	**-**	-	-	-	-	-

*Escherchia coli*	**-**	-	-	-	**-**	-	-	-	-	-

*Salmonella*	**-**	-	-	-	**-**	-	-	-	-	-

The bactericidal level was estimated by measuring the size of inhibition zone of the indicator strain. Insensitivity (-), low sensitivity (+: Diameter of inhibition < 15 mm), high sensitivity (++: Diameter of inhibition between 15 et 20 mm)

The minimum inhibition concentration (MIC) of **Tyrosol**, **TyC_8_**, **TyC_10 _**and **TyC_12 _**for *S. aureus, S. xylosus *and *B. cereus *was also investigated. Table 4 shows that **TyC_10 _**has the most potent effect. It exhibits the lowest MIC values towards the three tested strains (3.12 μg/ml against *B. cereus*, and 12.5 μg/ml against staphylococcus strains). The obtained MIC values of **TyC_8 _**were 12.5 μg/ml against *B. cereus *and 25 μg/ml against staphylococcus strains, while MIC values of **TyC_12 _**were estimated to more than 50 μg/ml against the three strains. Finally, **Ty **does not exhibit any antimicrobial effect up to 5 mg/ml.

**Table 4:** Minimum inhibitory concentrations (MIC) of Tyrosol (**Ty**) and Tyrosyl esters (**TyC_8_**, **TyC_10 _**and **TyC_12_**) for three microbial strains after 24 h of incubation at 37°C.

Compounds	MIC (μg/mL)		
	S. *aureus*	S. *xylosus*	B.*cereus*

Ty	ND	ND	ND

TyC_8_	25	25	12.5

TyC_10_	12.5	12.5	3.1

TyC_12_	50	50	> 100

ND*: Without effect up to 5 mg/ml.

### Antileishmanial activity

Tyrosol and its lipophilic derivatives were screened for their leishmanicidal activity. The screening was carried out using two *Leishmania *species: *L. major *GLC94 [23] and *L. infantum *LV50 [24]. As shown in Table 5 only three tyrosyl derivatives **TyC_8_**, **TyC_10 _**and **TyC_12 _**were effective against both *Leishmania *species while either tyrosol or short and long chain derivatives have no leishmanicidal activity. Interestingly, the three effective derivatives showed a higher activity against the *L. major *promastigotes compared to that obtained against *L. infantum *promastigote. Indeed, IC_50 _values were approximately two times higher against the former than the later. This indicates that *L. major *parasites are more sensitive to these compounds than *L. infantum *ones. The most effective compound is **TyC_10 _**which showed an IC_50 _of 19.21 μg/ml and 38.73 μg/ml against *L. major *and *L. infantum *respectively. On the other hand, **TyC_8 _**showed a moderate activity of 38.09 μg/ml and 62.8 μg/ml against *L. major *and *L. infantum *respectively. Finally, **TyC_12 _**was the less active compound of the three derivatives which showed an IC_50 _of 60.34 μg/ml and 157.6 μg/ml against the dermotropic and the visceraotropic spp respectively.

**Table 5:** IC_50 _activities of tyrosol and its acyl chain derivatives against *L. major *and *L. infantum *parasite species evaluated by the MTT assay

Compounds	Ty	**TyC**_**2**_	**TyC**_**3**_	**TyC**_**8**_	**TyC**_**10**_	**TyC**_**12**_	**TyC**_**16**_	**TyC**_**18**_	**TyC**_**18:1**_
IC50 (μg/ml) *L. major*	ND	ND	ND	**38.09**	**19.21**	**60.34**	ND	ND	ND

IC50 (μg/ml) *L. infantum*	ND	ND	ND	**62.8**	**38.73**	**157.6**	ND	ND	ND

* ND: Without effect up to 400 μg/ml

## Discussion

The biological activity of the lipophilic tyrosyl derivatives is at yet not well understood. After tyrosyl fatty acid ester derivatives production by a chemoselective enzymatic esterification of tyrosol we investigated in this study, and for the first time, the relationship of the carbon number of these tyrosyl derivatives and their antimicrobial and antileishmanial activities.

**Figure 2:**
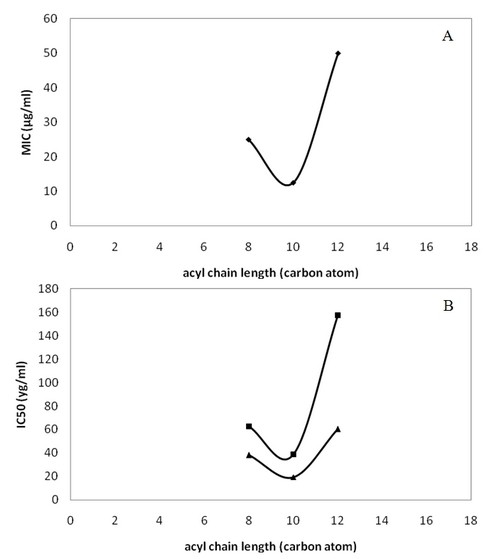
**Acyl chain length of each tyrosyl derivatives plotted relatively to (A) MIC values obtained on staphylococcus strains (black diamonds) or to (B) IC_50 _values obtained on *L. major *(black diamonds) or *L. infantum *(black triangle) parasites**.

As showed in Figure 2, when MIC and IC_50 _values are plotted against the acyl chain length for the tyrosyl derivatives, a parabolic shape with a maximum of 10 carbon atoms (**TyC_10_**) can be observed. This effect is probably due the surfactant effectiveness of the derivatives. Indeed, Lucas et al, [25] have observed in emulsion system that when the surfactant effectiveness is plotted against the acyl chain length for the tyrosol and hydroxytyrosol ester series, a parabolic shape with around 8-10 carbon atoms (caprylate and decanoate tyrosyl derivatives) indicating an adequate surfactant properties. Hence, these compounds could be considered as antioxidant surfactants. We can hypothesize that the antimicrobial and antileishmanial activities have a linear dependency to the surfactant property of a given tyrosyl derivative. Others [26] reported, in a study performed with cellular system (human fibroblasts), that the relationship of the interaction of the chlorogenic acid alkyl esters derivatives with biological membranes is quasi-parabolic, which means that the efficiency of the interaction of such compounds with biological membranes raises concomitantly to the increase of their hydrophobic parts up to a certain length and then begins to diminish. This effect was described for the first time in 1939 when compiling a combination of studies related to an homologous series of compounds [27]. Sometimes named the parabolic case, this effect is now known under the name of cut-off effect. Others [28] reported that this effect is a general phenomenon observed in various biological and toxic activities with practically every amphiphilic homologous series tested so far.

In the light of these results, caprylate, capriate and laurate tyrosyl esters could be promising agents in transdermal therapeutic systems to control the drug release and cutaneous absorption as it was investigated by Cso'ka et al, [29] for sucrose fatty acid ester delivery. Indeed, these authors reported that among laurate, myristate, palmitate and stearate sucrose tested as drug delivery agents, only medium fatty acid chain length (sucrose laurate) increased the amount of released drug about 10 times.

## Conclusion

Fatty acid with different chain lengths (from **C_2 _**to **C_18:1_**) have been used to synthesize tyrosyl derivatives using Novozyme 435 in order to obtain a wide hydrophilic-lipophilic phenolic compounds. From all the tested derivatives for their antimicrobial and antileishmanial activities, only medium chain derivatives (**TyC_8_, TyC_10 _**and **TyC_12_**) exhibited good antimicrobial and antileishmanial activities; maximum MIC and IC_50 _values are observed with **TyC_10_**. These results open up potential applications to use medium tyrosyl derivatives surfactants, antioxidants, antimicrobial and antileishmanial compounds in cosmetic, food and pharmaceutical industries.

## Materials and methods

### Materials

Tyrosol and deuterated chloroform (CDCl_3_) were purchased from Fluka (Switzerland), n-hexane from Prolabo (Paris, France) and ethyl acetate and proprionic acid from Pharmacia (Uppsala, Sweden). Caprylic, capric, palmitic and stearic, oleic acids and 2-methyl-2-propanol were purchased from Fluka (Germany). Lipase from *Candida antarctica *(Novozyme 435) was from Sigma Aldrish (Germany).

### Esterification reactions

Production of tyrosyl acetate (**TyC_2_**) was performed as previously reported by Aissa et al, [17]. Tyrosyl lipophilic esters (**TyC_3 _**to **TyC_18:1_**) were prepared by direct esterification of tyrosol by different fatty acids in screw-capped flasks. Tyrosol (20 mg) was dissolved in 4 ml equivalent volume ratio of 2-methyl-2-propanol/n-hexane. The fatty acid concentration was adjusted to obtain tyrosol/fatty acid molar ratio of eight. The mixture was stirred at 45°C in an orbital shaker at 200 rpm and in the presence of 20 mg of lipase. Control reactions in the absence of lipase were also realized. Aliquots from the mixture reaction were withdrawn at 72 h of incubation and filtered to be used for HPLC analysis. The conversion yield of tyrosyl derivatives was calculated as the ratio of number of moles of tyrosol converted per total number of tyrosol.

### HPLC Analysis

The identification and the conversion yield of tyrosyl derivatives were carried out by HPLC analysis. It was performed using a Dionex apparatus composed of an LC-10ATvp pump and an SPD-10Avp detector. The column used is a C-18 (4.6 × 250 mm; Shimpack VP-ODS), maintained at 35°C. The flow rate used was 1.5 mL/min. The mobile phase used was 0.05% acetic acid in water (A) versus 0.1% acetic acid in acetonitrile (B) for a total running time of 20 min and the following proportions of solvent B were used for the elution: 0-3 min: 10-30%; 3-5 min: 30-90%; 5-18 min: 90% and 18-20 min: 90-10%.

### Purification and identification of Tyrosyl esters

The reaction mixture resulting from the esterification of tyrosol with the different fatty acids contains a mixture of tyrosyl ester and residual substrates. After removal of the enzyme by centrifugation at 8000 rpm for 15 min, the mixture reaction was dried under nitrogen and 100 mg was taken up in 1 mL of Hexane. The purification of esters was achieved by chromatography on a silica gel 60 column (Merck) (25 cm × 2 cm), previously equilibrated in hexane. Elution was carried using Hexane/Diethyl ether/acetic acid mixtures (50:48:2). The collected solvent fractions were analyzed by TLC using the same mobile phase. The color appeared through evaporated iodine. Purified fractions were pooled and solvents were evaporated at 40°C under vacuum.

NMR spectra were recorded on a Bruker A-300, spectrophotometer operating at 300 MHz (^1^H) and 125,75 MHz (^13^C). For these experiments, samples were dissolved in deuterated chloroforme (CDCL_3_) and obtained data are described in Tables 2 and 3.

### Antibacterial activity

#### Diffusion well method

The bactericidal effects of tyrosol and its acyl esters were tested against several Gram positive and Gram negative bacteria on LB medium agar plate by agar spot assay [30]. The antibacterial activity was checked by well diffusion method [31]. Briefly, bacteria (previously pre-incubated 12 h in LB medium) were cultivated in LB medium at 37°C for 3 h. A basal layer of LB containing 16 g/l agar, was cooled in Petri dishes. When plates were dried, 10 ml of soft LB (8 g/l agar) containing 10^7 ^cells were overlaid. Wells were then punched in the agar plate and filled with 20 μl of each sample (20 mg/ml). After 24 h of incubation at 37°C, the zone of growth inhibition was measured to determine the level of bactericidal effect (Table 3).

Several bacteria strains were used: i.e., *Staphyloccocus *(*S*.) *aureus, S. epidermidis, Bacillus *(*B*.) *cereus, B. subtilis, Micrococcus *(*M*.) *Luteus, Enterococcus *(*E*.) *faecalis, E. faecium, Enterobacter *(*E*.) *cloacae, Brevibacterium *(*B*.) *flavum, Pseudomonas *(*P*.) *Aeruginosa, Salmonella, Klebsielle *(*K*.) *pneumonia *and *Echerichia *(*E*.) *coli*.

#### Determination of the minimum inhibitory concentration (MIC)

The Minimal Inhibitory Concentrations (MICs) of tyrosol and its derivatives against the tested microorganisms were determined by the broth microdilution method [32]. All tests were performed in LB, supplemented with ethanol (with 0.2% as a highest final concentration). *S. aureus, S. xylosus and B. cereus *strains were cultured overnight at 37°C in LB. Test strains were suspended in LB (The initial absorbance measured at 600 nm was approximately adjusted to 0.2 Optical density (OD) value). Geometric dilutions ranging from 100 μg/ml to 1.5 μg/ml of **TyC_8_**, **TyC_10 _**and **TyC_12 _**and 5 mg/ml to 0.625 mg/ml of tyrosol were prepared in 96-well microtiter plate, including one growth control (LB+ethanol). Plates were incubated under normal atmospheric conditions at 37°C for 24 h. Absorbance was then measured at 600 nm and MICs values were determined as the lowest tyrosol derivatives'concentrations inhibiting visible growth of bacterial strains. Tests were performed in duplicates.

### Parasite culture

*L. major *(MHOM/TN/95/GLC94 [23], and *L. infantum *(MHOM/TN/94/LV50 [24] strains isolated from Tunisian patients were used within this study. Promastigotes were cultured in solid medium at 26°C, and then progressively adapted to a complete medium composed of RPMI 1640 and 10% of Fetal Calf Serum (FCS). With a starting concentration of 3 × 10^6 ^parasites/ml, the stationary phase, where parasites are at their infective metacyclic forms, was reached after 6 days of culture.

### Parasite treatment

Promastigote parasites were washed twice with RPMI 1640, counted and dispatched at 10^7^parasites/well in the complete medium. Parasites were then incubated for 24 h in the presence of serially diluted concentrations of tyrosyl derivatives (ranging from 3.125 to 400 μg/ml). Negative controls correspond to parasites cultured in the absence of the tyrosyl derivatives and parasites with the elution buffer only. Tests were performed in duplicates.

### Leishmanicidal activity

The effects of tyrosyl derivatives on *Leishmania *promastigotes were evaluated by the MTT assay as described by Dutta *et al*., [33]. After treatment with tyrosyl derivative solutions, microtitre plates were centrifuged at 1700 g for 10 min and supernatants were removed and replaced with the same volume of 1 mg/ml of MTT freshly dissolved in PBS. Plates were then incubated overnight at room temperature and centrifuged at 2500 g. Formazan salt formed inside the parasite mitochondries was solubilized by discarding supernatants and adding SDS 10% for 2 h at 37°C in the dark. Absorbance was measured at 540 nm using an ELISA plate reader. OD of each treated sample was compared to those grown without extracts. Each assay was performed in duplicate and independent experiments were realized at least twice.

## List of abbreviations

**TyC_2_: **Tyrosyl acetate; **TyC_3_: **Tyrosyl propionate; **TyC_8_: **Tyrosyl caprylate; **TyC_10_: **Tyrosyl capriate; **TyC_12_: **Tyrosyl laurate; **TyC_16_: **Tyrosyl palmitate; **TyC_18_: **Tyrosyl stearate; **TyC _18:1_: **Tyrosyl oleate; **NMR**: Nuclear magnetic resonance; **CDCL_3_**: deuterated chloroforme **MIC: **Minimum inhibitory concentration; **IC_50_: **The half maximal inhibitory concentration; **GLC94**: *Leishmania major*; **LV50**: *Leishmania infantum*; **FCS**: Fetal Calf Serum; **RPMI**: Roswell Park Memorial Institute; **MTT**: bromure de 3-(4,5-dimethylthiazol-2-yl)-2,5-diphenyl tetrazolium; **PBS**: phosphate buffered saline.

## Competing interests

The authors declare that they have no competing interests.

## Authors' contributions

**IA **carried out all the studies, analyzed the data and drafted the manuscript. **RMS **carried out the antileishmanial activity, **MB**: helped with the NMR analysis. **DA **and **SS **helped with the discussion of the data and the correction of the manuscript. **YG **participated in the study design and helped to draft the manuscript. All authors have read and approved the final manuscript.
